# Oxidative Stress, Prooxidants, and Antioxidants: The Interplay

**DOI:** 10.1155/2014/761264

**Published:** 2014-01-23

**Authors:** Anu Rahal, Amit Kumar, Vivek Singh, Brijesh Yadav, Ruchi Tiwari, Sandip Chakraborty, Kuldeep Dhama

**Affiliations:** ^1^Department of Veterinary Pharmacology and Toxicology, Uttar Pradesh Pandit, Deen Dayal Upadhayay Pashu Chikitsa Vigyan Vishwa Vidyalaya Evam Go-Anusandhan Sansthan (DUVASU), Mathura 281001, India; ^2^Department of Veterinary Microbiology and Immunology, Uttar Pradesh Pandit, Deen Dayal Upadhayay Pashu Chikitsa Vigyan Vishwa Vidyalaya Evam Go-Anusandhan Sansthan (DUVASU), Mathura 281001, India; ^3^Department of Animal Husbandry, Kuchaman, Rajasthan 341508, India; ^4^Department of Veterinary Physiology, Uttar Pradesh Pandit, Deen Dayal Upadhayay Pashu Chikitsa Vigyan Vishwa Vidyalaya Evam Go-Anusandhan Sansthan (DUVASU), Mathura 281001, India; ^5^Animal Resources Development Department, Pt. Nehru Complex, Agartala 799006, India; ^6^Division of Pathology, Indian Veterinary Research Institute, Izatnagar, Bareilly 243122, India

## Abstract

Oxidative stress is a normal phenomenon in the body. Under normal conditions, the physiologically important intracellular levels of reactive oxygen species (ROS) are maintained at low levels by various enzyme systems participating in the *in vivo *redox homeostasis. Therefore, oxidative stress can also be viewed as an imbalance between the prooxidants and antioxidants in the body. For the last two decades, oxidative stress has been one of the most burning topics among the biological researchers all over the world. Several reasons can be assigned to justify its importance: knowledge about reactive oxygen and nitrogen species production and metabolism; identification of biomarkers for oxidative damage; evidence relating manifestation of chronic and some acute health problems to oxidative stress; identification of various dietary antioxidants present in plant foods as bioactive molecules; and so on. This review discusses the importance of oxidative stress in the body growth and development as well as proteomic and genomic evidences of its relationship with disease development, incidence of malignancies and autoimmune disorders, increased susceptibility to bacterial, viral, and parasitic diseases, and an interplay with prooxidants and antioxidants for maintaining a sound health, which would be helpful in enhancing the knowledge of any biochemist, pathophysiologist, or medical personnel regarding this important issue.

## 1. Introduction

Man and animals are exposed to a large number of biological and environmental factors like alterations in feed and husbandry practices, climatic variables, transportation, regrouping, the therapeutic and prophylactic activities, various stressors, and so forth. The ability of the man and animal to fight against these factors is important for maintenance of their health and productivity. Today, the entire world is witnessing an upsurge in chronic health complications like cardiovascular disease, hypertension, diabetes mellitus, different forms of cancer, and other maladies. Medical surveys suggest that diet may serve as a potential tool for the control of these chronic diseases [1, 2]. Regular chewing of tobacco along with inadequate diet is the most prominent finding to mortality due to lung cancer in USA [3]. Diets rich in fruit and vegetables have been reported to exert a protective effect against a variety of diseases, particularly the cardiovascular disease and cancer [4–10]. The primary nutrients thought to provide protection afforded by fruit and vegetables are the antioxidants [11, 12]. In an analysis, Potter [13] reviewed 200 epidemiological studies, the majority of which showed a protective effect of increased fruit and vegetable intake and concluded that the high content of polyphenolic antioxidants in fruits and vegetables is probably the main factor responsible for the beneficial effects. This awareness has led to a tremendous increase in the proportion of fruits and vegetables rich in antioxidant molecules on the dining table in the last two decades, but still the risk of chronic health problems refuses to decline, rather it upsurged with an enhanced vigour, giving rise to a very important question—why? If the health associated problems are due to oxidative stress and the dietary constituents are potent antioxidants, then the question of problem arrival should not be there. What happens when these antioxidants reach the body tissues of interest or are there other factors still to be unrevealed?

## 2. Stress

The term “stress” has been used in physics since unknown time as it appears in the definition of Hooke's law of 1658, but its first use in the biological science dates back to Sir Hans Selye's letter to the Editor of Nature in 1936. At that time, it was not accepted, but later on, after the famous address of Hans Selye at the prestigious College of France, it received approval among scientific community, but defining stress again troubled Selye over several years. Today, stress can be defined as a process of altered biochemical homeostasis produced by psychological, physiological, or environmental stressors [14]. Any stimulus, no matter whether social, physiological, or physical, that is perceived by the body as challenging, threatening, or demanding can be labeled as a stressor. The presence of a stressor leads to the activation of neurohormonal regulatory mechanisms of the body, through which it maintains the homeostasis [14]. The overall physiological impact of these factors and the adaptation ability of the body determine the variations in growth, development, productivity, and health status of the animals [15–17]. These alterations can be viewed as a consequence of general adaptation syndrome as postulated by Hans Selye [18] and usually return to their normal status once the stimulus has disappeared from the scene. Strong and sustained exposure to stress [16, 19, 20] may result in higher energy negative balance and may ultimately result in reduction in adaptation mechanisms, increase in the susceptibility to infection by pathogens, decline in productivity, and finally a huge economical loss [16, 19, 21].

Many of us puzzle between distress, stress, and oxidative stress. Distress differs from stress, which is a physiological reaction that can lead to an adaptive response [22]. Distress is comparatively difficult to define and generally refers to a state in which an animal cannot escape from or adapt to the external or internal stressors or conditions it experiences resulting in negative effects upon its well-being [22]. Stress leads to adaptation but distress does not. Stress is a commonly used term for oxidative stress. Any alteration in homeostasis leads to an increased production of these free radicals, much above the detoxifying capability of the local tissues [23]. These excessive free radicals then interact with other molecules within cells and cause oxidative damage to proteins, membranes, and genes. In this process they often create more free radicals, sparking off a chain of destruction. Oxidative damage has been implicated in the cause of many diseases such as cardiovascular diseases, neuronal degeneration, and cancer and has an impact on the body's aging process too. An altered response to the therapeutic agents has also been observed [12]. External factors such as pollution, sunlight, and smoking also trigger the production of free radicals.

**Figure 1:**
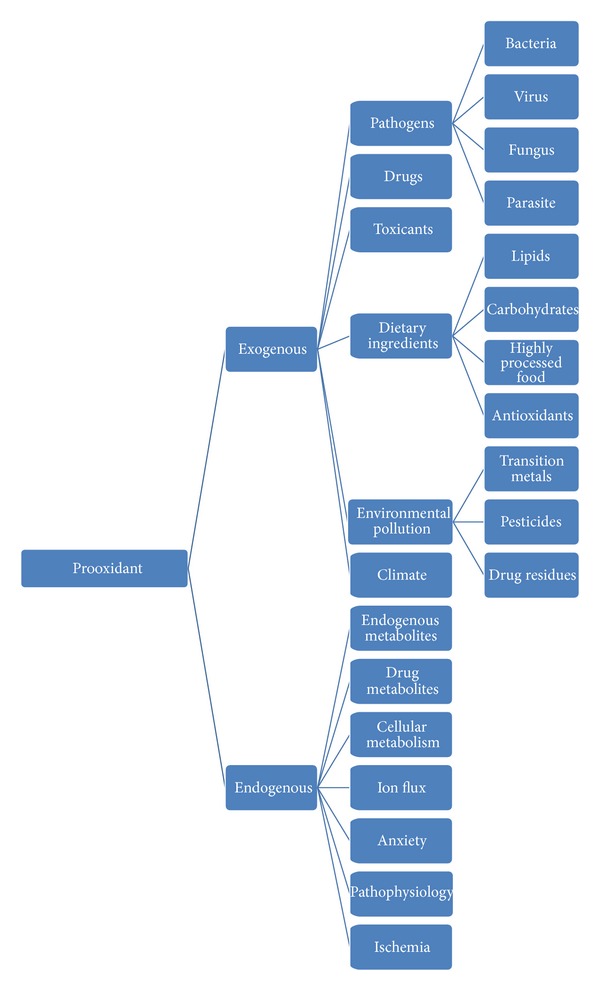
General classification of prooxidants.

Most importantly, stress is one of the basic etiologies of disease [24]. It can have several origins like environmental extremes for example, cold, heat, hypoxia, physical exercise or malnutrition (Figure 1).

On the basis of duration and onset, stress might be acute and chronic stress. The stress due to exposure of cold or heat is generally of acute type and is released with the removal of cause. Similarly, stress due to physical exercises or complete immobilization [25] is also acute in nature. The nutritional and environmental stresses, where the causes persist for a longer period of time, are chronic stress.

### 2.1. Cold Stress

Cold stress is evident whenever the temperature falls below 18°C and the body experiences severe cold related illness and permanent tissue damage. An acute cold stress (−20°C for 4 hours) in rats causes profound reduction in contraction amplitude with an increase in heart rate in the isolated heart preparations [26]. The decrease in amplitudes is associated with inadequate ATP formation. While changing perfusion of poststress isolated heart, myocardial rigidity further slows down and this seemed to be associated with activated glycolysis. There are no signs of cardiomyocytic lesion after cold stress. Reduced coronary flow is the only abnormal effect of acute cold stress under these conditions. High cardiac resistance to the damaging effect of cold is likely to be related to increased processes of glycolysis and glycogenolysis in the cardiomyocytes. The activity of succinate dehydrogenase also gets elevated indicating the influence of cold stress on the Krebs cycle [27]. Coronary blood flow is also reduced and later on results in an altered basophils activity in the myocardium [28].

### 2.2. Physical Exercise and Stress

Health benefits of regular physical exercise are undebatable. Both resting and contracting skeletal muscles produce reactive oxygen and nitrogen species (ROS, RNS). Low physiological levels of ROS are generated in the muscles to maintain the normal tone and contractility, but excessive generation of ROS promotes contractile dysfunction resulting in muscle weakness and fatigue [29]. This is perhaps the reason why intense and prolonged exercise results in oxidative damage to both proteins and lipids in the contracting muscle fibers [30].

Regular exercise induces changes in both enzymatic and nonenzymatic antioxidants in the skeletal muscle. Furthermore, oxidants can modulate a number of cell signaling pathways and regulate the expression of multiple genes in eukaryotic cells. This oxidant-mediated change in gene expression involves changes at transcriptional, mRNA stability, and signal transduction levels. The magnitude of exercise-mediated changes in superoxide dismutase (SOD) activity of skeletal muscle increases as a function of the intensity and duration of exercise [31, 32]. Mild physical activity increases nuclear factor-*kappa* B (NF-*κ*B) activity in the muscle of rats as well as the gene expression for manganese superoxide dismutase (MnSOD) and endothelial nitric oxide synthase (eNOS) [33].

### 2.3. Chronic Stress

Chronic stress significantly alters limbic neuroarchitecture and function and potentiates oxidative stress [25] and emotionality in rats [34]. Chronic restraining of laboratory animals has been found to increase aggression, potentiate anxiety, and enhance fear conditioning [34]. Chronic immobilization induces anxiety behavior and dendritic hypertrophy in the basolateral amygdala, which persist beyond a recovery period. Restraint of rats causes increased mucin release, as measured by [3H] glucosamine incorporation and goblet cell depletion, prostaglandin E2 (PGE2) secretion, and mast cell activation in colonic explants [35]. Upregulation of the neurotensin precursor mRNA in the paraventricular nucleus of the hypothalamus after immobilization has also been reported [36]. Neurotensin stimulates mucin secretion from human colonic goblet cell line by a receptor mediated mechanism [37].

### 2.4. Nutritional Stress

Nutrition is one of the most significant external etiologies for oxidative stress including its characteristics, type and quality, ratio of the various nutrients, dietary balance with regard to protein, carbohydrates, fats, macro- and trace elements, and so forth. Feed exercises a considerable influence over the physiological condition and thus the homeostasis of the animal body [16, 19, 38–42]. Feeding of endogenous or exogenous antioxidants can sensitively regulate glycolysis and the Warburg effect in hepatoma cells [43]. Fasting induces an increase in total leukocytes counts, eosinophils, and metamyelocytes in the blood profile, accompanied by a decrease in the basophils and monocytes, a typical “stress leukogram” produced in the animal body due to the increased endogenous production of cortisol from the adrenal glands during oxidative stress [16, 19, 38–42]. The leukocytosis with neutrophilia associated with fasting may be a consequence of an inflammatory reaction, caused by the direct action of ammonia on the rumen wall [38, 44]. The monocytopenia may be a result of adaptation and defense mechanism undergoing in the body and leads to higher susceptibility to pathogens [21, 45].

Nutritional stress causes adrenal gland hyperfunction and, thus, an increased release of catecholamines in the blood, with a simultaneous inhibition of the production of insulin in the pancreas [20, 38, 46–48]. The process of glycogenolysis is observed in the first 24 hours of fasting [20, 39, 46–49]. Thereafter, gluconeogenesis from amino acid precursors and lipolysis from glycerol, as well as from lactate through the Cori cycle, maintain a regular supply of glucose. Lactate gets transformed into pyruvate and participates in the gluconeogenesis along with the deaminated amino acids. The increased production of catecholamines (epinephrine and dopamine) owing to fasting results in peripheral vasoconstriction and redistribution in blood which is expressed as erythrocytosis, leukocytosis, and neutrophilia [47].

### 2.5. Hypoxic Stress

Hypoxia is known to stimulate mitochondria to release ROS (mROS). Under hypoxic conditions, mitochondria participate in a ROS burst generated at complex III of the electron transport chain [50]. Hypoxia and reoxygenation result in reversible derangement of ATPase and architecture of mitochondrial membrane. Cardiac hemodynamic parameters, which decline immediately under hypoxic conditions, recover during reoxygenation [51], but the biochemical and histopathological studies provide a complicated pattern [52]. High CAT (carboxyatractyloside) sensitivity of the ATPase is observed at 5 min of hypoxia. The initial phase in hypoxic perfusion (<15 min) exhibits a steep increase of ADP contents and ATPase activities and a drastic fall of ATP/ADP ratios in mitochondria, as well as in tissues. Furthermore, the number of ATPase particles visible at the inner aspect of mitochondrial membrane decreases. During the second phase of hypoxic perfusion (from 30 min onwards), the count of ATPase particles visible at the inner mitochondrial membrane further decreases. ATPase activities fluctuate, retaining close contact with the membrane during hypoxia. The mitochondrial ultrastructural damage becomes more evident. High-energy phosphates reserves of myocardium could help myocardial cells to maintain their structural integrity [52]. ATP/ADP ratios attain values of almost 1. During reoxygenation (after 30 min of hypoxia), the levels of mitochondrial adenine nucleotides, oxidative phosphorylation rate, and respiratory control index increase within 20 min and then slightly decline again. The ATP/ADP ratio is diminished in the course of reoxygenation. ATPase activity also decreases within 20 min of reoxygenation and the ADP/O ratio reaches control values. The ATPase activity gains its highest sensitivity towards catalase at 10 min of reoxygenation attaining a value similar to that of 5 min of hypoxic perfusion.

## 3. Stress and Well-Being

Each cell in the human body maintains a condition of homeostasis between the oxidant and antioxidant species [53]. Up to 1–3% of the pulmonary intake of oxygen by humans is converted into ROS [54]. Under conditions of normal metabolism, the continuous formation of ROS and other free radicals is important for normal physiological functions like generation of ATP, various catabolic and anabolic processes and the accompanying cellular redox cycles. However, excessive generation of free radicals can occur due to endogenous biological or exogenous environmental factors, such as chemical exposure, pollution, or radiation.

There are ROS subgroups: free radicals such as superoxide radicals (O_2_^•−^) and nonradical ROS such as hydrogen peroxide (H_2_O_2_) [55]. The primary free radicals generated in cells are superoxide (O_2_^•^) and nitric oxide (NO). Superoxide is generated through either incomplete reduction of oxygen in electron transport systems or as a specific product of enzymatic systems, while NO is generated by a series of specific enzymes (the nitric oxide synthases). Both superoxide and NO are reactive and can readily react to form a series of other ROS and RNS.

Generally, mitochondria are the most important source of cellular ROS where continuous production of ROS takes place [55]. This is the result of the electron transport chain located in the mitochondrial membrane, which is essential for the energy production inside the cell [56, 57]. Additionally, some cytochrome 450 enzymes are also known to produce ROS [58].

## 4. Biochemical Basis of Stress

Several endogenous cells and cellular components participate in initiation and propagation of ROS (Table 1) [59–63].

All these factors play a crucial role in maintenance of cellular homeostasis. A stressor works by initiating any of these mechanisms. Oxidative stress occurs when the homeostatic processes fail and free radical generation is much beyond the capacity of the body's defenses, thus promoting cellular injury and tissue damage. This damage may involve DNA and protein content of the cells with lipid peroxidation of cellular membranes, calcium influx, and mitochondrial swelling and lysis [60, 63, 64]. ROS are also appreciated as signaling molecules to regulate a wide variety of physiology. It was first proposed in the 1990s when hydrogen peroxide was shown to be required for cytokine, insulin, growth factor, activator protein-1 (AP-1), and NF-*κ*B signaling [65, 66]. The role of hydrogen peroxide in promoting phosphatase inactivation by cysteine oxidation provided a likely biochemical mechanism by which ROS can impinge on signaling pathways [67]. The role of ROS in signaling of cytochrome c mediated apoptosis is also well established [68]. ROS can cause reversible posttranslational protein modifications to regulate signaling pathways. A typical example of the beneficial physiological role of free radicals is a molecule of nitric oxide (NO). NO is formed from arginine by the action of NO-synthase (NOS) [69]. NO is produced by constitutive NOS during vasodilating processes (eNOS) or during transmission of nerve impulses (nNOS). In the presence of stressors, NO is produced by catalytic action of inducible NOS (iNOS) and is at higher concentrations [70–72]. NO can cause damage to proteins, lipids, and DNA either directly or after reaction with superoxide, leading to the formation of the very reactive peroxynitrite anion (nitroperoxide) ONOO– [73–75].

Lipid peroxidation of polyunsaturated lipids is one of the most preferred markers for oxidative stress. The product of lipid peroxidation, malondialdehyde, is easily detected in blood/plasma and has been used as a measure of oxidative stress. In addition, the unsaturated aldehydes produced from these reactions have been implicated in modification of cellular proteins and other constituents [76]. The peroxidized lipid can produce peroxy radicals and singlet oxygen.

## 5. Physiological Role of Stress

Stress has a significant ecological and evolutionary role and may help in understanding the functional interactions among life history traits [77–79]. Stress leads to a number of physiological changes in the body including altered locomotor activity and general exploratory behavior. The physiological role of ROS is associated with almost all of the body processes, for example, with reproductive processes [80]. Since under physiological conditions a certain level of free radicals and reactive metabolites is required, complete suppression of FR formation would not be beneficial [81]. One further beneficial example of ROS seen at low/moderate concentrations is the induction of a mitogenic response.

Stress leads to activation of hypothalamic-pituitary-adrenal axis. The increased endogenous catecholamine release has been observed in cold environmental conditions. The activity of succinate dehydrogenase also gets elevated indicating the influence of ROS as evident in cold environmental conditions [27]. Coronary blood flow is reduced and an altered basophils activity in the myocardium is also observed [28].

Free radicals play an irreplaceable role in phagocytosis as one of the significant microbicidal systems [82], or in several biochemical reactions, for example, hydroxylating, carboxylating, or peroxidating reactions, or in the reduction of ribonucleotides [83]. At present, free radicals and their metabolites are assumed to have important biomodulating activities and a regulatory ability in signal transduction process during transduction of intercellular information [83].

Among the reactive oxygen species, H_2_O_2_ best fulfills the requirements of being a second messenger [84]. Its enzymatic production and degradation, along with its functional requirement for thiol oxidation, facilitate the specificity for time and place that are required in signaling. Both the thermodynamic and kinetic considerations support that among different possible oxidation states of cysteine, formation of sulfenic acid derivatives or disulfides can be applicable as thiol redox switches in signaling. H_2_O_2_ readily diffuses across biological membranes, and so it is well-suited as a diffusible messenger [85, 86].

In the presence of transition metals such as iron or copper, H_2_O_2_ can give rise to the indiscriminately reactive and toxic hydroxyl radical (HO^•^) by Fenton chemistry. Increasing evidence indicates that H_2_O_2_ is a particularly an intriguing candidate as an intracellular and intercellular signaling molecule because it is neutral and membrane permeable [84, 87].

Specifically, H_2_O_2_ can oxidize thiol (–SH) of cysteine residues and form sulphenic acid (–SOH), which can get glutathionylated (–SSG), form a disulfide bond (–SS–) with adjacent thiols, or form a sulfenyl amide (–SN–) with amides [88]. Each of these modifications modifies the activity of the target protein and thus its function in a signaling pathway. Phosphatases appear to be susceptible to regulation by ROS in this manner, as they possess a reactive cysteine moiety in their catalytic domain that can be reversibly oxidized, which inhibits their dephosphorylation activity [67]. Specific examples of phosphatases known to be regulated in this manner are PTP1b, PTEN, and MAPK phosphatases [89]. Any emotional stress leads to a decrease in sympathetic outflow as well as energy production of the tissues [27].

## 6. Oxidative Stress

The harmful effect of free ROS and RNS radicals causing potential biological damage is termed oxidative stress and nitrosative stress, respectively [90–92]. This is evident in biological systems when there is either an excessive production of ROS/RNS and/or a deficiency of enzymatic and nonenzymatic antioxidants. The redox stress/oxidative stress is a complex process. Its impact on the organism depends on the type of oxidant, on the site and intensity of its production, on the composition and activities of various antioxidants, and on the ability of repair systems [93].

The term “ROS” includes all unstable metabolites of molecular oxygen (O_2_) that have higher reactivity than O_2_ like superoxide radical (O_2_^•^) and hydroxyl radical (HO^•^) and nonradical molecules like hydrogen peroxide (H_2_O_2_). These ROS are generated as byproduct of normal aerobic metabolism, but their level increases under stress which proves to be a basic health hazard. Mitochondrion is the major cell organelle responsible for ROS production [50, 57]. It generates ATP through a series of oxidative phosphorylation processes. During this process, one- or two-electron reductions instead of four electron reductions of O_2_ can occur, leading to the formation of O_2_^•^ or H_2_O_2_, and these species can be converted to other ROS. Other sources of ROS may be reactions involving peroxisomal oxidases [94], cytochrome *P*-450 enzymes [95], NAD (P)H oxidases [96], or xanthine oxidase [97].

## 7. Oxidative Stress and Diseases

**Figure 2:**
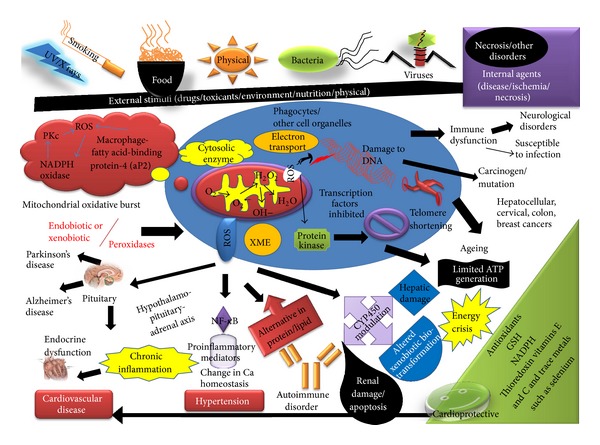
Oxidative stress and disease development.

Today the world is experiencing a rise in age related chronic health diseases like cardiovascular disorders, cancer, and so forth and their associated negative health impacts and mortality/casualty [98–101]. Some metabolic diseases like diabetes are also associated with an enhanced level of lipoperoxidation (Figure 2).

The central nervous system (CNS) is extremely sensitive to free radical damage because of a relatively small total antioxidant capacity. The ROS produced in the tissues can inflict direct damage to macromolecules, such as lipids, nucleic acids, and proteins [102]. The polyunsaturated fatty acids are one of the favored oxidation targets for ROS. Oxygen-free radicals, particularly superoxide anion radical (O_2_^•−^), hydroxyl radical (OH^•−^), and alkylperoxyl radical (^•^OOCR), are potent initiators of lipid peroxidation, the role of which is well established in the pathogenesis of a wide range of diseases. Once lipid peroxidation is initiated, a propagation of chain reactions will take place until termination products are produced. Therefore, end products of lipid peroxidation, such as malondialdehyde (MDA), 4-hydroxy-2-nonenol (4-HNE), and F2-isoprostanes, are accumulated in biological systems. DNA bases are also very susceptible to ROS oxidation, and the predominant detectable oxidation product of DNA bases *in vivo* is 8-hydroxy-2-deoxyguanosine. Oxidation of DNA bases can cause mutations and deletions in both nuclear and mitochondrial DNA. Mitochondrial DNA is especially prone to oxidative damage due to its proximity to a primary source of ROS and its deficient repair capacity compared with nuclear DNA. These oxidative modifications lead to functional changes in various types of proteins (enzymatic and structural), which can have substantial physiological impact. Similarly, redox modulation of transcription factors produces an increase or decrease in their specific DNA binding activities, thus modifying the gene expression.

Among different markers of oxidative stress, malondialdehyde (MDA) and the natural antioxidants, metalloenzymes Cu, Zn-superoxide dismutase (Cu, Zn-SOD), and selenium dependent glutathione peroxidase (GSHPx), are currently considered to be the most important markers [103–106]. Malondialdehyde (MDA) is a three-carbon compound formed from peroxidized polyunsaturated fatty acids, mainly arachidonic acid. It is one of the end products of membrane lipid peroxidation. Since MDA levels are increased in various diseases with excess of oxygen free radicals, many relationships with free radical damage were observed.

Cu, Zn-SOD is an intracellular enzyme present in all oxygen-metabolizing cells, which dismutates the extremely toxic superoxide radical into potentially less toxic hydrogen peroxide. Cu, Zn-SOD is widespread in nature, but being a metalloenzyme, its activity depends upon the free copper and zinc reserves in the tissues. GSHPx, an intracellular enzyme, belongs to several proteins in mammalian cells that can metabolize hydrogen peroxide and lipid hydroperoxides.

## 8. Oxidative Stress and Altered Immune Function

The relationship between oxidative stress and immune function of the body is well established. The immune defense mechanism uses the lethal effects of oxidants in a beneficial manner with ROS and RNS playing a pivotal role in the killing of pathogens. The skilled phagocytic cells (macrophages, eosinophils, heterophils), as well as B and T lymphocytes, contain an enzyme, the nicotinamide adenine dinucleotide phosphate (NADPH) oxidase [107, 108], which is responsible for the production of ROS following an immune challenge. At the onset of an immune response, phagocytes increase their oxygen uptake as much as 10–20 folds (respiratory burst). The O^•−^ generated by this enzyme serves as the starting material for the production of a suite of reactive species. Direct evidence also certifies production of other powerful prooxidants, such as hydrogen peroxide (H_2_O_2_), hypochlorous acid (HOCl), peroxynitrite (ONOO–), and, possibly, hydroxyl (OH^•^) and ozone (O_3_) by these cells. Although the use of these highly reactive endogenous metabolites in the cytotoxic response of phagocytes also injures the host tissues, the nonspecificity of these oxidants is an advantage since they take care of all the antigenic components of the pathogenic cell [109].

Several studies have demonstrated the interdependency of oxidative stress, immune system, and inflammation. Increased expression of NO has been documented in dengue and in monocyte cultures infected with different types of viral infections. Increased production of NO has also been accompanied with enhancement in oxidative markers like lipid peroxidation and an altered enzymatic and nonenzymatic antioxidative response in dengue infected monocyte cultures [110]. More specifically, the oxygen stress related to immune system dysfunction seems to have a key role in senescence, in agreement with the oxidation/inflammation theory of aging. Moreover, it has been revealed that reduced NADPH oxidase is present in the pollen grains and can lead to induction of airway associated oxidative stress. Such oxidative insult is responsible for developing allergic inflammation in sensitized animals. There is triggering of production of interleukin (IL)-8 along with proinflammatory cytokines, namely, tumor necrosis factor (TNF)-alpha and IL-6. There is initiation of dendritic cell (DC) maturation that causes significant upregulation of the expression of cluster of differentiation (CD)-80, 86 and 83 with a slight overexpression of CD-40 in the membrane. So altogether, innate immunity locally may be alleviated due to oxidative stress induced by exposure to pollen. This in turn helps in participation to initiate adaptive immune response to pollen antigens [111]. 

The immune status directly interplays with disease production process. The role of physical and psychological stressors contributes to incidences and severity of various viral and bacterial infections. Both innate as well as acquired immune responses are affected by the altered IFN-*γ* secretion, expression of CD14, production of the acute-phase proteins, and induction of TNF-*α*. Fatal viral diseases produce severe oxidative stress (OS) leading to rigorous cellular damage. However, initiation, progress, and reduction of damages are governed by the redox balance of oxidation and antioxidation. The major pathway of pathogenesis for cell damage is via lipid peroxidation particularly in microsomes, mitochondria, and endoplasmic reticulum due to OS and free radicals [112, 113]. All the factors responsible for the oxidative stress directly or indirectly participate in immune system defense mechanism. Any alteration leading to immunosuppression can trigger the disease production (Table 2).

## 9. Oxidative Stress and Incidence of Autoimmune Diseases

Oxidative stress can induce production of free radicals that can modify proteins. Alterations in self-antigens (i.e., modified proteins) can instigate the process of autoimmune diseases [114, 115]. Under oxidative stress, cells may produce an excess of ROS/RNS which react with and modify lipids and proteins in the cell [116]. The end products of these reactions may be stable molecules such as 3-chlorothyrosine and 3-nitrotyrosine that may not only block natural biotransformations of the tyrosine like phosphorylation but also change the antigenic profile of the protein. The oxidative modification of the proteins not only changes the antigenic profile of latter but also enhances the antigenicity as well [117]. There exist several examples of autoimmune diseases resulting from oxidative modifications of self-proteins, namely, systemic lupus erythematosus (60 kD Ro ribonucleoprotein) [118], diabetes mellitus (high molecular weight complexes of glutamic acid decarboxylase) [119], and diffuse scleroderma (oxidation of beta-2-glycoprotein) [120, 121].

Moreover, oxidative stress poses an additional threat to the target tissues as in the case of insulin-producing beta cells in the islet of Langerhans [122]. To add to this, autoimmune diseases often occur only in a single tissue irrespective of the fact that other tissues also contain the same antigen but perhaps lack the level of oxidative stress required to initiate the process. This pathological autoreactivity targeted towards redox-modified self-antigens and diagnostic assays designed to measure its cross-reactivity to normal self-antigens further complicate the detection of autoimmune diseases [123]. In the development of autoimmune disease pathogenesis, there is possibly role of psychological stress along with major hormones that are related to stress. It is thereby presumed that the neuroendocrine hormones triggered by stress lead to dysregulation of the immune system ultimately resulting in autoimmune diseases by alteration and amplification of production of cytokine [124].

## 10. Oxidative Stress and Altered Susceptibility to Bacterial, Viral, and Parasitic Infections

All pathogens, irrespective of their classification, bacterial, viral, or parasitic, with impaired antioxidant defenses show increased susceptibility to phagocytic killing in the host tissues, indicating a microbicidal role of ROS [80]. Vice versa to this, different studies have proven that individuals deficient in antioxidative mechanism are more susceptible to severe bacterial and fungal infections as in case of HIV infections [125]. Reactive species are important in killing pathogens but as a negative side effect can also injure the host tissues (immunopathology). This is particularly apparent during chronic inflammation, which may cause extensive tissue damage with a subsequent burst in oxidative stress [126]. The production of free radicals involves macrophages and neutrophils to combat the invading microbes. The whole of the process is performed in host cells during the activation of phagocytes or the effect of bacteria, virus, parasites, and their cell products reactivity with specific receptors. The multicomponent flavoprotein NADPH oxidase plays vital role in inflammatory processes by catalyzing the production of superoxide anion radical O_2_^−^ and excessive production of reactive oxygen species (ROS) leads to cellular damage. These cellular damages in general lead to altering immune response to microbes and ultimately altered susceptibility to bacterial, viral, and parasitic infections [127].

## 11. Oxidative Stress and Increase in Levels of Incidence and Prevalence of Various Malignancies

Carcinogenesis can be defined as a progressive erosion of interactions between multiple activating and deactivating biological activities (both immune and nonimmune) of host tissue resulting in progressive loss of integrity of susceptible tissues. The primitive steps in development of cancer, mutation, and ageing are the result of oxidative damage to the DNA in a cell. A list of oxidized DNA products has been identified currently which can lead to mutation and cancer. Major change noticed due to ROS caused DNA damage is the break in the DNA strand, due to the alterations in the purine or pyrimidine ring [56, 66]. Alongside with ROS other redox metals also play a critical role in development of ageing, mutation, and tumour [128]. In regular cellular mechanism, free radicals scavenger vitamin E, C and glutathione along with enzymes like catalase, peroxidases, and superoxide dismutase control the mechanism of DNA repair. These damages are either in the form of single strand breaks (SSBs), double strand breaks (DSBs), or oxidatively generated clustered DNA lesions (OCDLs). Irregular repair or absence of repair of damaged DNA due to OS might lead to mutagenesis and genetic transformation along with alteration in apoptotic pathway [129].

Oxidative stress produced due to unresolved and persistent inflammation can be a major factor involved in the change of the dynamics of immune responses. These alterations can create an immunological chaos that could lead to loss of architectural integrity of cells and tissues ultimately leading to chronic conditions or cancers [130]. Oxidative stress is reported to be the cause of induction of allergies, autoimmune or neurodegenerative diseases along with altered cell growth, chronic infections leading to neoplasia, metastatic cancer, and angiogenesis [131]. Damage to the cellular components such as proteins, genes, and vasculature is behind such alterations. Moreover, further accumulation of confluent, useless, and complex cells causes additional oxidative stress and maintains continuous activation of immune system and unanswered inflammation [132]. Tissue necrosis and cellular growth are stimulated by coexpression of inflammatory mediators due to oxidative stress-induced altered activity of the cells of the immune system. Such changes of tissue function are mainly responsible for autoimmune, neurodegenerative, and cancerous conditions [133, 134]. Various factors produced due to oxidative stress along with excessively produced wound healing and apoptotic factors, namely, TNF, proteases, ROSs, and kinases, actively participate in tumor growth and proliferation. These factors are also required for the membrane degradation, invasion of neighboring tissues, and migration of tumor cells through vasculature and lymphatic channels for metastasis [135–137]. The incidences of thyroid cancers have increased in the last decades worldwide which is most likely due to exposure of human population in mass to radiation causing increased free radical generation [138].

## 12. Oxidative Stress and Aging

Aging is an inherent mechanism existing in all living cells. There is a decline in organ functions progressively along with the age-related disease development. The two most important theories related to ageing are free radical and mitochondrial theories, and these have passed through the test of time. There is claim by such theories that a vicious cycle is generated within mitochondria wherein reactive oxygen species (ROS) is produced in increased amount thereby augmenting the damage potential [139]. Oxidative stress is present at genetic, molecular, cellular, tissue, and system levels of all living beings and is usually manifested as a progressive accumulation of diverse deleterious changes in cells and tissues with advancing age that increase the risk of disease and death [140]. Recent studies have shown that with age, ROS levels show accumulation in major organ systems such as liver, heart, brain, and skeletal muscle [141–145] either due to their increased production or reduced detoxification. Thus, aging may be referred to as a progressive decline in biological function of the tissues with respect to time as well as a decrease in the adaptability to different kinds of stress or briefly an overall increase in susceptibility to diseases [146]. Oxidative stress theory is presently the most accepted explanation for the aging which holds that increases in ROS lead to functional alterations, pathological conditions and other clinically observable signs of aging, and finally death [147]. No matter whether mitochondrial DNA damage is involved or electron transport chain damage is responsible for aging, modulation of cellular signal response to stress or activation of redox-sensitive transcriptional factors by age-related oxidative stress causes the upregulation of proinflammatory gene expression, finally leading to an increase in the ROS levels [146].

## 13. Genomic Evidences of the Stress-Disease Development Interrelationship

Persistent oxidative stress due to altered inflammation acts as precancerous state of host cells leading to the initiation of genetic mutations, genetic errors, epigenetic abnormalities, wrongly coded genome, and impaired regulation of gene expression [148]. Events like methylation of nucleic acid, binding of DNA proteins, formation and binding of histone proteins, function of repair, and enzyme mediated modifications are sensitive to free radicals formed during oxidative stress [149]. These events involved in epigenetic modification and telomere-telomerase pathways can induce mutations of suppressor genes [150]. The suppression of genes alters somatic maintenance and repair leading to altered proliferative control of gene expression, polymorphism, and contact inhibition regulation and telomere shortening [151]. The activation or progressive transformation of cancer cells is also augmented by inactivated or mutated suppressor gene pathways. Moreover, abnormal DNA methylation of CpG and various enzymatic pathways influence inflammation and carcinogenesis [152]. The theory of modus operandi for pathogenesis of vitiligo, a multifactorial polygenic disorder, also moves around autoimmune, cytotoxic, oxidant-antioxidant, and neural mechanisms [153].

Lipid originated atherosclerosis also involves endoplasmic reticulum (ER) stress in macrophages. ER stress mitigation with a chemical chaperone leads to massive protection against macrophage associated lipotoxic death. This causes prevention of expression of macrophage-fatty acid-binding protein-4 (aP2). There is also an increase in the phospholipid (rich in monounsaturated fatty acid as well as bioactive lipids) production due to absence of lipid chaperones. There is also further impact of aP2 on metabolism of lipid in the macrophages. The stress response in ER is also mediated by key lipogenic enzymes upregulation in the liver [154]. Similarly, OS due to alcohol toxicity triggers the release of certain cytokines to activate collagen gene expression in liver stellate cells leading to progression of liver fibrosis [155].

## 14. Proteomic Evidences of the Stress-Disease Development Interrelationship

Oxidative damages mediated by free radicals lead to protein modification and ultimately cellular damages and disease pathogenesis. There lies equilibrium between the antioxidants level and cellular prooxidants under normal conditions of physiology. But when there is occurrence of environmental factors or stressors, there exists an imbalance in the homeostasis which is in favour of prooxidants. This results in the oxidative stress phenomenon [156]. An antioxidant deficiency can also result in oxidative stress leading to generation of reactive oxygen or nitrogen species in excess [157]. The 20S proteosome often removes the proteins that are damaged oxidatively. The proteosome systemic defects result in increased levels of proteins that are oxidatively modified along with development of neurotoxicity [158–162]. For instance, oxidation of nucleic acid and protein along with peroxidation of lipid is highest and most severe in the hippocampus of the brain, which is involved in the processing of memory along with cognitive function [158, 159]. Such study is strongly suggestive of the fact that a primary event in the Alzheimer's disease development is an oxidative stress [163]. These alterations and modifications in proteomes elicit antibodies formation in diseases like rheumatoid arthritis (RA), diabetes mellitus (DM), and systemic lupus erythematosus [164].

## 15. Assessment of Oxidative Stress

The concentration of different reductant-oxidant markers is considered an important parameter for assessing the prooxidant status in the body tissues [83]. Several indicators of *in vivo* redox status are available, including the ratios of GSH to GSSG, NADPH to NAPD^−^, and NADH to NAD^−^, as well as the balance between reduced and oxidized thioredoxin. Out of these redox pairs, the GSH-to-GSSG ratio is thought to be one of most abundant redox buffer systems in mammalian species [93]. A decrease in this ratio indicates a relative shift from a reduced to an oxidized form of GSH, suggesting the presence of oxidative stress at the cellular or tissue level. In aging, an age-related shift from a redox balance to an oxidative profile is observed which results in a reduced ability to buffer ROS that are generated in both “normal” conditions and at times of challenge [23, 83, 93, 147]. Thus, a progressive shift in cellular redox status could potentially be one of the primary molecular mechanisms contributing to the aging process and accompanying functional declines.

## 16. Prooxidants

Prooxidant refers to any endobiotic or xenobiotic that induces oxidative stress either by generation of ROS or by inhibiting antioxidant systems. It can include all reactive, free radical containing molecules in cells or tissues. Prooxidants may be classified into several categories (Table 3).

Some of the popular and well known antioxidant flavonoids have been reported to act as prooxidant also when a transition metal is available [165]. These have been found to be mutagenic *in vitro* [102, 166–168]. The antioxidant activities and the copper-initiated prooxidant activities of these flavonoids depend on their structures. The OH substitution is necessary for the antioxidant activity of a flavonoid [169]. Flavone and flavanone, which have no OH substitutions and which provide the basic chemical structures for the flavonoids, show neither antioxidant activities nor copper-initiated prooxidant activities. The copper initiated prooxidant activity of a flavonoid also depends on the number of free OH substitutions on its structure [170]. The more the OH substitutions, the stronger the prooxidant activity. *O*-Methylation and probably also other *O*-modifications of the flavonoid OH substitutions inactivate both the antioxidant and the prooxidant activities of the flavonoids.

The antioxidant activity of quercetin has been found to be better than its monoglucosides in a test system wherein lipid peroxidation was facilitated by aqueous oxygen radicals [171]. Luteolin has also proved to be a significantly stronger antioxidant than its two glycosides [172].

Flavonoids generally occur in foods as *O*-glycosides with sugars bound at the C3 position. Methylation or glycosidic modification of the OH substitutions leads to inactivation of transition metal-initiated prooxidant activity of a flavonoid.

The protection provided by fruits and vegetables against diseases, including cancer and cardiovascular diseases, has been attributed to the various antioxidants, including flavonoids, contained in these foods. Flavonoids, such as quercetin and kaempferol, induce nuclear DNA damage and lipid peroxidation in the presence of transition metals. The *in vivo* copper-initiated prooxidant actions of flavonoids and other antioxidants including ascorbic acid and *α*-tocopherol are generally not considered significant, as copper ion will be largely sequestered in the tissues, except in case of metal toxicity. The prevention of iron-induced lipid peroxidation in hepatocytes by some flavonoids including quercetin is well known [49, 173].

## 17. Antioxidants

To counteract the harmful effects taking place in the cell, system has evolved itself with some strategies like prevention of damage, repair mechanism to alleviate the oxidative damages, physical protection mechanism against damage, and the final most important is the antioxidant defense mechanisms. Based on the oxidative stress related free radical theory, the antioxidants are the first line of choice to take care of the stress. Endogenous antioxidant defenses include a network of compartmentalized antioxidant enzymic and nonenzymic molecules that are usually distributed within the cytoplasm and various cell organelles. In eukaryotic organisms, several ubiquitous primary antioxidant enzymes, such as SOD, catalase, and several peroxidases catalyze a complex cascade of reactions to convert ROS to more stable molecules, such as water and O_2_. Besides the primary antioxidant enzymes, a large number of secondary enzymes act in close association with small molecular-weight antioxidants to form redox cycles that provide necessary cofactors for primary antioxidant enzyme functions. Small molecular-weight nonenzymic antioxidants (e.g., GSH, NADPH, thioredoxin, vitamins E and C, and trace metals, such as selenium) also function as direct scavengers of ROS. These enzymatic and nonenzymatic antioxidant systems are necessary for sustaining life by maintaining a delicate intracellular redox balance and minimizing undesirable cellular damage caused by ROS [83]. Endogenous and exogenous antioxidants include some high molecular weight (SOD, GPx, Catalse, albumin, transferring, metallothionein) and some low molecular weight substances (uric acid, ascorbic acid, lipoic acid, glutathione, ubiquinol, tocopherol/vitamin E, flavonoids).

Natural food-derived components have received great attention in the last two decades, and several biological activities showing promising anti-inflammatory, antioxidant, and anti-apoptotic-modulatory potential have been identified [17, 174, 175]. Flavonoids comprise a large heterogeneous group of benzopyran derivatives present in fruits, vegetables, and herbs. They are secondary plant metabolites and more than 4000 molecular species have been described. Flavonoids exert a positive health effect in cancer and neurodegenerative disorders, owing to their free radical-scavenging activities [169]. One of the most abundant natural flavonoids present in a large number of fruits and vegetables is quercetin (3,5,7,3′,4′, pentahydroxyflavone) which prevents oxidative injury and cell death by scavenging free radicals, donating hydrogen compound, quenching singlet oxygen, and preventing lipid peroxidation or chelating metal ions [176]. Red wines also have a high content of phenolic substances including catechin and resveratrol [177], which are responsible for the antioxidant action, anti-inflammatory, antiatherogenic property, oestrogenic growth-promoting effect, and immunomodulation. Recently, the potential of resveratrol as an antiaging agent in treating age-related human diseases has also been proven.

## 18. Interplay of Antioxidative and Prooxidative Role of Antioxidants

Ascorbic acid has both antioxidant and prooxidant effects, depending upon the dose [178]. Low electron potential and resonance stability of ascorbate and the ascorbyl radical have enabled ascorbic acid to enjoy the privilege as an antioxidant [179, 180]. In ascorbic acid alone treated rats, ascorbic acid has been found to act as a CYP inhibitor. Similar activity has also been observed for other antioxidants-quercetin [181] and chitosan oligosaccahrides [182], which may act as potential CYP inhibitors. Specifically, Phase I genes of xenobiotic biotransformation, namely, CYP1A1, CYP2E1, and CYP2C29, have been previously reported to be downregulated in female rats in the presence of a well known antioxidant, resveratrol [183]. The antioxidant and prooxidant role of ascorbic acid in low (30 and 100 mg/kg body weight) and high doses (1000 mg/kg body weight), respectively, has also been reported in case of ischemia induced oxidative stress [178]. The *in vivo* prooxidant/antioxidant activity of betacarotene and lycopene has also been found to depend on their interaction with biological membranes and the other co-antioxidant molecules like vitamin C or E [184]. At higher oxygen tension, carotenoids tend to lose their effectiveness as antioxidants. In a turn around to this, the prooxidant effect of low levels of tocopherol is evident at low oxygen tension [185].

Moreover, *α*-lipoic acid exerts a protective effect on the kidney of diabetic rats but a prooxidant effect in nondiabetic animals [186]. The prooxidant effects have been attributed to dehydroxylipoic acid (DHLA), the reduced metabolite of *α*-lipoic acid owing to its ability to reduce iron, initiate reactive sulfur-containing radicals, and thus damage proteins such as alpha 1-antiproteinase and creatine kinase playing a role in renal homeostasis [186]. An increase in *α*-lipoic acid and DHLA-induced mitochondrial and submitochondrial O_2_^−^ production in rat liver [187] and NADPH-induced O_2_^−^ and expression of p47phox in the nondiabetic kidney has also been observed [186].

Withaferins, the pharmacological molecules of* Withania somnifera *L. Dunal (commonly known as Ashwagandha), have been used safely for thousands of years in Ayurvedic medicine practice for the treatment of various disorders [188–191]. In the last 5–10 years, numerous reports revealed the proapoptotic effects of withaferins [192–198]. Withaferins can also initiate apoptosis and prevent metastasis of breast carcinomas under the influence of interleukin-6-induced activation and transcription [176] and prove to be of tremendous clinical benefit to human patients. In accordance to these reports, recently withaferin-induced apoptosis has been found to be mediated by ROS production due to inhibition of mitochondrial respiration [199].

Use of ginseng and *Eleutherococcus senticosus* is thought to increase the body's capacity to tolerate external stresses, leading to increased physical or mental performance [200]. Although an extensive literature documenting adaptogenic effects in laboratory animal systems exists, results from human clinical studies are conflicting and variable [200–202]. However, there is evidence that extracts of ginseng and *Eleutherococcus *sp. can have an immunostimulatory effect in humans, and this may contribute to the adaptogen or tonic effects of these plants [200, 203]. From laboratory studies, it has been suggested that the pharmacological target sites for these compounds involve the hypothalamus-pituitary-adrenal axis due to the observed effects upon serum levels of adrenocorticotropic hormone and corticosterone [202]. However, it should also be noted that the overall effects of the ginsenosides can be quite complex due to their potential for multiple actions even within a single tissue [201].

The flavonoids present in ginkgo extracts exist primarily as glycosylated derivatives of kaempferol and quercetin [203–205]. These flavonoid glycosides have been shown to be extremely effective free radical scavengers [201, 202, 205]. It is believed that the collective action of these components leads to a reduction in damage and improved functioning of the blood vessels [200, 202].

Depending on the type and level of ROS and RNS, duration of exposure, antioxidant status of tissues, exposure to free radicals and their metabolites leads to different responses—increased proliferation, interrupted cell cycle, apoptosis, or necrosis [165]. A typical example is a hydrophilic antioxidant, ascorbic acid (vitamin C). Ascorbic acid reacts with free radicals to produce semidehydro- or dehydroascorbic acids (DHA). DHA is then regenerated by antioxidant enzymes present in the organism (semidehydroascorbic acid reductase and dehydroascorbic acid reductase) back to the functional ascorbate. In the presence of ions of transition metals, ascorbic acid reduces them and it gets oxidized to DHA. Hydrogen peroxide formed in the reaction further reacts with reduced metal ions leading to generation of hydroxyl radical through Fenton type reaction. Iron ions practically never occur in the free form in the tissues; therefore, the occurrence of Fenton type reaction *in vivo* is not likely.

Recently, toxicity of ascorbic acid has also been attributed to its autooxidation. Ascorbic acid can be oxidized in the extracellular environment in the presence of metal ions to dehydroascorbic acid, which is transported into the cell through the glucose transporter (GLUT). Here it is reduced back to ascorbate. This movement of electrons changes the redox state of the cell influencing gene expression.

## 19. Conclusions

Oxidative stress is nothing but the imbalance between oxidants and antioxidants in favor of the oxidants which are formed as a normal product of aerobic metabolism but during pathophysiological conditions can be produced at an elevated rate. Both enzymatic and nonenzymatic strategies are involved in antioxidant defense, and antioxidant efficacy of any molecule depends on the cooxidant. Well proven free radical scavengers can be prooxidant unless linked to a radical sink. Moreover, as the free radicals share a physiological as well as pathological role in the body, the same antioxidant molecule just due to its free radical scavenging activity may act as disease promoter, by neutralizing the physiologically desired ROS molecules, and as disease alleviator by removing the excessive levels of ROS species. The importance of several vitamins like vitamin A and tocopherols as well as carotenes, oxycarotenoids, and ubiquinols in their lipid phase has been understood in recent years. Low molecular mass antioxidant molecules that include nuclear as well as mitochondrial matrices, extracellular fluids, and so forth have been studied vividly to understand how they accelerate the body defense significantly. Protection from the influence of oxidants being an important issue has become the centre of attraction of the scientists and various research groups in recent years to understand the mechanism of action of various antioxidants present in herbs as well as fruits and vegetables that can act as antiaging agents as well. There has been ever increasing knowledge in the role of oxygen derived prooxidants and antioxidants that play crucial role in both normal metabolism and several clinical disease states. Advances in the field of biochemistry including enzymology have led to the use of various enzymes as well as endogenous and exogenous antioxidants having low molecular weight that can inhibit the harmful effect of oxidants. Still much research works are needed to understand the antioxidant status of any organ that is susceptible to oxidative stress induced damage particularly the involvement of genetic codes and gene protein interaction. Understanding of genetic alterations and molecular mechanism is certainly helping out to reveal the interaction of free radicals and their role in proteomics, genomics and disease development process. Moreover, the prooxidant or antioxidant behavior of the universally accepted antioxidant molecules is now duly expressed in term of dependence upon the actual molecular conditions prevailing in the tissues.

Nevertheless, other environmental factors like oxygen tension, concentration of transition metals along with their redox status will also be a deciding factor. Thus, it can be concluded that a thorough knowledge of biochemistry and general chemistry will help the researchers to explore more the interplay between oxidative stress, prooxidants, and antioxidants.

## Figures and Tables

**Table 1:** Endogenous mediators of oxidative stress.

Leakage of free radicals	Membrane-bound enzymes	NADPH oxidase
	Electron transport systems	Mixed function oxidases

Activation of oxygen	Soluble cell constituents	Transition metals, thiol containing proteins, quinine derivatives, epinephrine, metalloproteins, hemeproteins, and flavoproteins
	Xenobiotic metabolizing enzymes	Cyt P_450_-dependent monooxygenases, Cyt *b*_5_, and NADPH-dependent cytochrome reductases

ROS generation/propagation	Soluble cytosolic enzymes	Xanthine oxidase, superoxide dismutase, catalase
	Phagocytic cells	Neutrophils, macrophages, and monocytes involved in inflammation, respiratory burst, and removal of toxic molecules
	Local ischemia	Damaged blood supply due to injury or surgery

**Table 2:** Deadly diseases that have got positive correlation to oxidative stress.

Sl. number	Disease	Organs involved	Etiology	References
(1)	Macular degeneration	Eyes	Reactive oxygen intermediates (ROI)	[206]
(2)	Diabetes	Multi-organ	Superoxide dismutase, catalase, glutathione reductase, glutathione peroxidase	[207]
(3)	Chronic fatigue	Multiorgan	C-reactive protein	[208]
(4)	Atherosclerosis	Blood vessels	Reduced NADPH oxidase system	[209]
(5)	Autoimmune disorders (systemic lupus erythematosus)	Immune system	R_*o*_ ribonucleoprotein	[118]
(6)	Neurodegenerative diseases (Alzheimer's and Parkinson's disease)	Brain	Reactive oxygen species (ROS)	[210]
(7)	Asthma	Lungs	ROS particularly H_2_O_2_	[211]
(8)	Rheumatoid and osteoarthritis	Joints	Radical oxygen species	[212]
(9)	Nephritis	Kidney	Glutathione transferase kappa (GSTK 1-1)	[213]
(10)	Melanoma	Skin	Pathophysiological processes including DNA damage and lipid peroxidation (LPO)	[214]
(11)	Myocardial infarction	Heart	Reactive oxygen species (ROS)	[215]

**Table 3:** Different classes of prooxidants and their common mechanism for development of oxidative stress.

Sl. number	Class	Examples	Mechanism
(1)	Drugs	Common over-the-counter drug like analgesic (paracetamol) or anticancerous drug (methotrexate)	ROS generation leading to alterations in macromolecules which finally can fatally damage the tissues mainly liver and kidney

(2)	Transition metals	Magnesium, iron, copper, zinc, and so forth	These metals induce Fenton reaction and Haber-Weiss reaction leading to generation of excessive ROS. Chronic magnesium is a classic prooxidant disease. The other can be hemochromatosis due to high iron levels or Wilson disease due to copper

(3)	Pesticide	BHC, DDT, and so forth	Stimulation of free radical production, induction of lipid peroxidation, alterations in antioxidant enzymes and the glutathione redox system

(4)	Physical exercise	Running, weight lifting	Relaxationcontraction of muscle involves production of ROS. Rigorous exercise leads to excessive ROS

(5)	Mental anxiety	Tension, apprehension	Imbalance in the redox system plays a role in neuroinflammation and neurodegeneration, mitochondrial dysfunction, altered neuronal signaling, and inhibition of neurogenesis

(6)	Pathophysiology	Local ischemia	Gives rise to increased ROS generation

(7)	Environmental factor	Extreme weather (heat, cold, thunderstorm)	During adaptation, mitochondrial membrane fluidity decreases which may disrupt the transfer of electrons, thereby increasing the production of ROS

(8)	Antioxidants	Ascorbic acid, vitamin E, polyphenols	Act as prooxidant under certain circumstances, for example, heavy metals
